# Translating Unconventional T Cells and Their Roles in Leukemia Antitumor Immunity

**DOI:** 10.1155/2021/6633824

**Published:** 2021-01-07

**Authors:** Nilberto Dias de Araújo, Fábio Magalhães Gama, Mateus de Souza Barros, Thaís Lohana Pereira Ribeiro, Fabíola Silva Alves, Lilyane Amorim Xabregas, Andréa Monteiro Tarragô, Adriana Malheiro, Allyson Guimarães Costa

**Affiliations:** ^1^Programa de Pós-Graduação em Imunologia Básica e Aplicada, Universidade Federal do Amazonas (UFAM), Manaus, AM 69067-005, Brazil; ^2^Diretoria de Ensino e Pesquisa, Fundação Hospitalar de Hematologia e Hemoterapia do Amazonas (HEMOAM), Manaus, AM 69050-001, Brazil; ^3^Programa de Pós-Graduação em Ciências Aplicadas à Hematologia, Universidade do Estado do Amazonas (UEA), Manaus, AM 69850-000, Brazil; ^4^Programa de Pós-Graduação em Medicina Tropical, Universidade do Estado do Amazonas (UEA), Manaus, AM 69850-000, Brazil; ^5^Instituto de Pesquisa Clínica Carlos Borborema, Fundação de Medicina Tropical Doutor Heitor Vieira Dourado (FMT-HVD), Manaus, AM 69040-000, Brazil

## Abstract

Recently, cell-mediated immune response in malignant neoplasms has become the focus in immunotherapy against cancer. However, in leukemia, most studies on the cytotoxic potential of T cells have concentrated only on T cells that recognize peptide antigens (Ag) presented by polymorphic molecules of the major histocompatibility complex (MHC). This ignores the great potential of unconventional T cell populations, which include gamma-delta T cells (*γδ*), natural killer T cells (NKT), and mucosal-associated invariant T cells (MAIT). Collectively, these T cell populations can recognize lipid antigens, specially modified peptides and small molecule metabolites, in addition to having several other advantages, which can provide more effective applications in cancer immunotherapy. In recent years, these cell populations have been associated with a repertoire of anti- or protumor responses and play important roles in the dynamics of solid tumors and hematological malignancies, thus, encouraging the development of new investigations in the area. This review focuses on the current knowledge regarding the role of unconventional T cell populations in the antitumor immune response in leukemia and discusses why further studies on the immunotherapeutic potential of these cells are needed.

## 1. Introduction

Leukemia comprises a heterogeneous group of hematological neoplasms, which can be classified into lymphoblastic or myeloid leukemias and divided into acute and chronic types, depending on the affected cell type, maturation stage, and blast count, respectively [1]. While acute leukemias are characterized by a deep block in hematopoietic differentiation and result in an overproduction of immature blasts, chronic leukemias are characterized by the excessive production of partially mature differentiated cells, for example, lymphocytes in chronic lymphocytic leukemia (CLL) and granulocytes in chronic myeloid leukemia (CML) [2, 3]. The hallmark of these neoplasms is the increase in leukemic cells (LCs) in the bone marrow (BM) and their release in the peripheral blood (PB) and in extramedullary sites [1].

The immunological mechanisms in patients with leukemia are not very well known. However, with the increasing advances in the field of immunotherapy, there has been great progress in research regarding the tumor microenvironment in leukemia. Studies have shown that LCs secrete factors that disrupt healthy BM niches, reprogramming and transforming them into “leukemic niches,” as well as inducing a disruption in balanced cytokine production, and favoring leukemic persistence and metastatic potential [4, 5]. However, despite the protumor microenvironment created by LCs, studies have reported that a specific immune response can be triggered and, therefore, contribute to the defense against the tumor, although not sufficient enough to control the neoplasia [6].

Several studies have described the immunotherapeutic potential of CD8 and CD4 T cells that recognize peptide antigens (Ag) presented by polymorphic major histocompatibility complex (MHC) class I and MHC class II molecules, respectively [7, 8]. Leaving aside, the populations of T cells are considered “unconventional,” which are also implicated in tumor immunity, although their role in them is not well understood. Collectively, these T cell populations differ from their conventional counterparts mainly in the way they recognize and respond to foreign molecules. Unlike MHC-reactive T cells, unconventional T cells generally show simplified patterns of the T cell antigen receptor (TCR) expression and usually target monomorphic Ag-presenting molecules and other ligands, where after their activation, they promote rapid and strong effector responses [9, 10]. These T cell populations include *γδ* T cells, NKT cells, and MAIT cells.

In this review, we describe the main characteristics of these T cell populations and explore their activities during the neoplastic process, as well as their relationship with the establishment of an antitumor immune response or tumor-favorable response, as described briefly in Table 1. A better understanding of the participation of these underexplored cells in tumor dynamics may provide a basis for the development of potential immunotherapeutic strategies in the field of leukemias.

## 2. Subsets of Unconventional T Cells in Antileukemic Response

### 2.1. Gamma-Delta (*γδ*) T Cells

*γδ* T cells are developed in the thymus during lymphopoiesis and, by the expression of the TCR (T-cell receptor), composed of gamma (*γ*) and delta (*δ*) chains [11]. These cells are capable of providing a potent and lasting immune response through innate and adaptive mechanisms and stand out for their recognition and destruction of several tumors, regardless of major histocompatibility complex (MHC) expression [12]. In addition, these cells constitute up to 10% of circulating CD3^+^ cells and are classified according to the genetic rearrangements of the *δ* chain of the TCR into three main subsets: V*δ*1, V*δ*2, and V*δ*3 (in humans) [13]. These lymphocytes are present in blood circulation, tissues, and mucosal membranes, which are strategic places to exercise their high cytotoxic power against infections and tumors [14].

**Figure 1:**
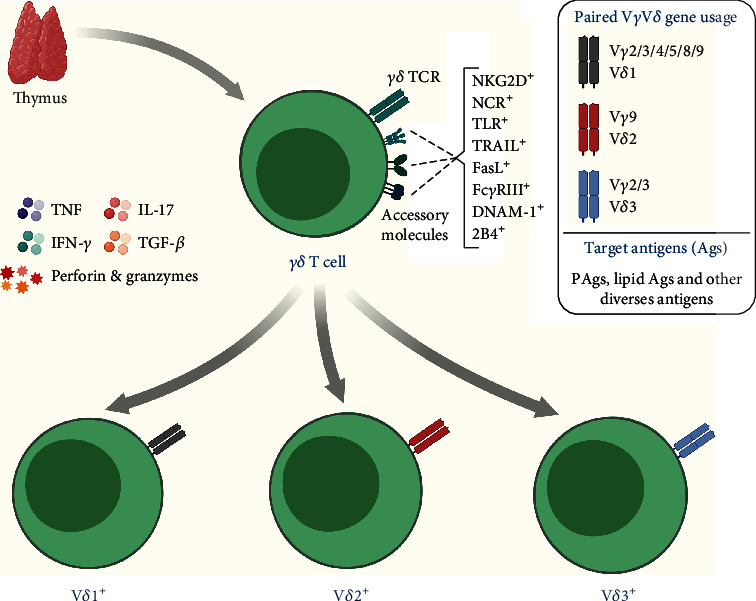
Overview of subpopulations, important receptors, and cytokines produced by *γδ* T cells. *γδ* T cells express some receptors that are essential for the tumor recognition and destruction, which gives a certain advantage when compared to other conventional T lymphocyte populations, either due to MHC independence or due to the high expression of the receptors mentioned in the image. The antileukemic recognition repertoire includes several molecules, such as NKG2D, NCRs, FasL, CD16, DNAM-1, and the TCR *γδ* itself.

*γδ* T cell lineage is evolutionarily conserved and has several pleiotropic functions. While these cells are naturally specialized in the secretion of proinflammatory mediators (Figure 1) [15], these lymphocytes can also adopt Th2-, Th9-, and Th17-like response phenotypes [16–18]. In the tumor microenvironment (TME), these lymphocytes express a diverse repertoire of recognition receptors that have received favorable prognostic value in several malignancies, including leukemia [19]. Based on several reports, it has been confirmed that *γδ* T cells are capable of triggering an immune response by direct and indirect mechanisms, for example, through the cytolytic synapse with the target cell, and the recruitment and stimulation of other immune cells necessary for the establishment of an antitumor response [12, 20].

These lymphocytes can induce neoplastic regression through cell-cell interaction or by secreting several soluble molecules (such as IFN-*γ* and TNF) that inhibit tumor expansion. These effector molecules induce an increase in the antitumor activity of other cytotoxic cells or positively regulate the expression of MHC-I by cancer cells [21]. In addition, *γδ* T cells stimulate somatic hypermutation and isotype switching in B cells [22–24] and possibly induce antibody-mediated immunity. Their effector functions also include the activation of macrophages and the recruitment and activation of CD8^+^ cytotoxic T cells and NK cells [25–28]. The immune role of these lymphocytes also includes stimulating the maturation of dendritic cells (DCs), and, in turn, DCs are able to potentiate their cytotoxic activity [29, 30]. Notably, cancer cells tend to express several stress-induced molecules or metabolic antigens that are recognized through *γδ* TCRs and accessory receptors, thereby, mediating a potent response against the tumor [31–34].

Without restrictions on MHC expression, *γδ* T cells recognize several antigens that are expressed in LCs, and generally include metabolic molecules and stress-induced molecules [32, 35–38]. Ligands, such as MIC-A/MIC-B and ULBPs, can be identified through the NKG2D receptor, which is expressed mainly in *γδ* T cells [34, 39]. Furthermore, some metabolites of the mevalonate pathway, known as phosphoantigens (pAgs), can be recognized directly through the TCRs and are highly regulated in LCs [40, 41]. In addition, other molecules assist in tumor recognition and possibly support the antileukemic response of *γδ* T cells, such as TLRs (toll-like receptors), DNAM-1 (DNAX Accessory Molecule-1), FasL (Fas ligand), Fc*γ*RIII, TRAIL (TNF-related apoptosis-inducing ligand), NCRs (Natural Cytotoxicity Receptors) such as NKp30, NKp44, and NKp46, and the 2B4 receptor [12, 36, 41–44].

V*δ*1^+^ cells, which express TCR chains V*γ*1 to V*γ*11, respond preferentially in skin tissues, intestinal epithelium, lung, spleen, and liver, where they play crucial roles in maintaining epithelial tissue [14, 45]. It is known that these lymphocytes patrol the several tissues in search of stressed cells, derived from infections and tumorigenesis, thus, maintaining tissue homeostasis [46]. For this, the secretion of Th1 and Th17 cytokines is essential for immune surveillance [12, 47–49].

These cells, although uncommon in peripheral blood (~ 10% of blood *γδ* T cells), have a high diversity of tumor recognition and have demonstrated great potential against LCs [36, 50, 51]. Correia et al. demonstrated that V*δ*1^+^ cells that expressed NCRs managed to destroy lymphoid and myeloid cancer cells through NKp30 and NKp44, which seemed to recognize antigens that are distinct from their classic ligands, such as the molecule B7-H6, which binds to NKp30 [36]. In the same study, the stable expression of these NCRs was associated with elevated levels of granzyme B and seemed to synergize greater cytotoxic activity against LCs [36]. Taking into account that in some leukemias, a high expression of members of the ULBP family is observed, as NKG2D ligands [52], Lança et al. demonstrated that the expression of ULBP1 in LCs is important for recognition by V*δ*1 cells [51]. ULBP3 was confirmed by Poggi et al. who presented similar findings [52]. Therefore, the data suggest possible immunological participation of these cells and indicate a significant contribution to the antileukemic immune response.

These cells can also recognize lipid or metabolic antigens that are presented through MHC class I-like molecules, such as CD1 and MR1 [46, 53]. These and other characteristics of V*δ*1 cells make them potential candidates for new immunotherapeutic approaches in several human tumors, and these have recently been explored in several experimental trials, including some against leukemia [54–56]. Although their roles are still poorly known, we know that V*δ*1 cells demonstrate important functions in antitumor activity, and these deserve to be highlighted since they present characteristics that are different to V*γ*9V*δ*2 cells, either due to their high expression of NCRs or the nonsusceptibility to activation-induced cell death (AICD) [57–59]. The roles of this subset of *γδ* T cells in the environment of bone marrow and peripheral blood *in vivo*, in the context of leukemia, still need further investigation regarding the ligands and receptors of recognition that are engaged during the immune response.

The V*δ*2^+^ subset, which pairs exclusively with the V*γ*9 TCR chain, responds mainly in the blood, where it recognizes pAgs derived from bacteria and cancer cells [60]. Once activated, these lymphocytes secrete effector molecules such as IFN-*γ*, TNF, perforins, and granzymes and exert important cytotoxic activities in peripheral blood against pathogens and tumors [12, 61]. These cells make up as much as 95% of blood *γδ* T cells [50, 62–64] and generally respond to a wide variety of pAgs, such as IPP (isopentenyl pyrophosphate) and HMB-PP (4-hydroxy-3-methyl-but-2-enylpyrophosphate), which are intermediates of the mevalonate pathway in eukaryotes and prokaryotes [65–67]. The recognition of these pAgs occurs in the context of the butyrophilin (BTN) family of molecules, such as BTN3A1 and BTN2A1 [68–71], which can be detected in LCs, and mediates a potent immune response that can be used therapeutically [72]. These molecules can be recognized directly through the *γδ* TCR and are capable of triggering a Th1-like response against the target cells [73, 74].

The mechanism for recognizing pAgs is not yet clear, although several studies have recently expanded the information about how *γδ* T cells identify these molecules. Recent reports have pointed out that pAgs recognition is mediated by BTN-like molecules, which are expressed in cancer cells [72, 75] and are able to modulate the responses of conventional *αβ* T cells [76–79], and most notably, of *γδ* T cells [68–71, 80, 81]. The dependent detection of pAgs by V*γ*9V*δ*2 cells involves the entire structure of the TCR, which interacts with BTN molecules through the V*γ*9 and V*δ*2 TCR domains. Among the various molecules that make up the BTN family, the proteins BTN3A1 and BTN2A1 synergize the presentation of pAgs to *γδ* T cells, binding directly to the TCR V*γ*9V*δ*2 [68, 70, 82, 83].

Previously, it was thought that the unit expression of BTN3A1 performed the activation of these lymphocytes alone [84], but it is now clear that the BTN2A1 protein acts as a critical factor in the activation of V*δ*2 cells [70]. For this to occur, it is necessary that pAgs bind to the intracellular domain (B30.2) of these proteins [85, 86]. After binding of pAgs, the intra- and extracellular domains of BTN3A1 and BTN2A1 undergo a conformational change [85–87] that allows the contact of the TCR V*γ*9 chain with the BTN2A1 molecule, sending activation signals to *γδ* T cells [68]. In addition, the involvement of other molecules during the pAgs detection mechanism cannot be ignored, as recent reports suggest the molecular collaboration of CDR3, periplakin, and GTPase RhoB in this process [70, 88, 89].

In addition to direct and TCR-dependent recognition, other accessory molecules possibly support the antileukemic activity of V*δ*2 cells against LCs, such as the DNAM-1 receptor that recognizes the PVR (poliovirus receptor) and Nectin-2 ligands, both expressed in LCs [41], and ULBP4 that can be recognized through NKG2D [90]. *γδ* T cells also express the 2B4 receptor (which recognizes the CD48 ligand), an accessory molecule that strengthens target effector interactions and is possibly related to increased cytotoxic activity against cancer cells [44]. One disadvantage of these V*δ*2^+^ cells is their strong propensity for AICD upon prolonged exposure to antigens and the polarization of these lymphocytes towards a tumor-promoting phenotype, which limits the persistence and efficiency of the immune response [57, 59].

Little data is available regarding the receptors and ligands involved in the events of innate and adaptive immunity mediated by V*δ*3^+^ cells, which express the TCR chains V*γ*2, V*γ*3, or V*γ*8 and are a specific subset that responds mainly in the liver [91, 92]. The frequency of this cell population is low in peripheral blood (~0.2% of total circulating T cells) though it is high in the liver and intestinal region [91, 92]. Studies have reported that V*δ*3^+^ cells are related to antiviral immunity, responding efficiently against cytomegalovirus [93], Epstein-Barr virus [94], hepatitis [95], and HIV infection [64]. So far, their roles in antitumor immunity are not clear, although some reports have identified an expansion of these lymphocytes in the peripheral blood of patients with leukemia [96]. Also, V*δ*3 cells secrete IFN-*γ*, express NKG2D, Fc*γ*RIII, and CD161, and appear to respond in a restricted way to CD1d, functionally resembling iNKT cells, which recognize and destroy CD1d^+^ target cells [24, 91]. Subsequently, it was identified that these cells also respond to ANX-2 (annexin-2) [97], but their role in leukemia is still unexplored, although it has been identified that these cells expand during tumor progression [96].

*γδ* T cells have an enormous potential to regulate local immunity and remodel the tumor niche [45, 98, 99]. The evidence discussed so far suggests that these cells realize various antitumor activities through the direct identification of LCs through the *γδ* TCR or accessory receptors and by secreting soluble effector molecules against the tumor. Despite this, emerging knowledge about the molecular and cellular interaction between these lymphocytes and cancer cells suggests that, like conventional *αβ* T cells, *γδ* T cells may possibly not be exempt from the immunosuppression established by the TME [100–103]. However, the lack of knowledge about these cells in the leukemic microenvironment makes it difficult to elaborate larger and more comprehensive discussions about the possible crosstalk between this population of lymphocytes and the LCs in the bone marrow compartment and in extramedullary sites.

In addition, other subsets of *γδ* T cells that express other variable TCR chains (TCR domains V*δ*5, V*δ*6, and V*δ*8) have been identified in other hematological malignancies, where, for example, these cells seemed to expand and respond to cancer cells in the blood of patients with lymphoma [104]. However, it is still unclear whether these cells respond against LCs and not have information available on the possible roles played by these *γδ* T cell subsets in leukemia antitumor immunity. In view of its enormous therapeutic applicability, further research is needed on the interaction of *γδ* T cells and their subsets in the leukemic microenvironment and on how this can impact the prognosis of patients.

### 2.2. Natural Killer T (NKT) Cells

Natural killer T cells (NKT) correspond to a population of innate-like T cells characterized by the expression of a TCR composed of alpha (*α*) and beta (*β*) chains similar to those of conventional T cells, in addition to specific surface markers of natural killer cells (NK), such as CD16^+^, CD56^+^, CD69^+^, CD161^+^, NKG2D^+^, and Ly49A^+^ [105, 106]. Another striking feature of these cells is their restriction to the CD1d molecule, which is an MHC class I-like molecule that presents lipid and glycolipid antigens [107–109].

NKT cells constitute approximately 0.001-1% of circulating lymphocytes (in humans) and are also present in the thymus, liver, intestine, and spleen [110, 111]. These cells are divided into two main subsets, the invariant natural killer T (iNKT) and natural killer T type II (NKTII) cells [112]. Both express the following transcription factors: T-bet [113, 114], PLZF [115], ROR*γ*t, GATA-3 [114], and NF-kB [116], which together grant high cellular plasticity and allow polarization for phenotypes of profile Th1, Th2, and Th17 [116–118]. In addition, iNKTs express the LEF-1 factor [119, 120], which is correlated with the regulation of the expression of the gene that encodes the CD1d molecule in antigen presenting cells (APCs). The LEF-1 factor also plays a crucial role as a regulator of the Wnt pathway, and it is possible that it influences the growth, development, differentiation, and functions of NKT cells [121].

**Figure 2:**
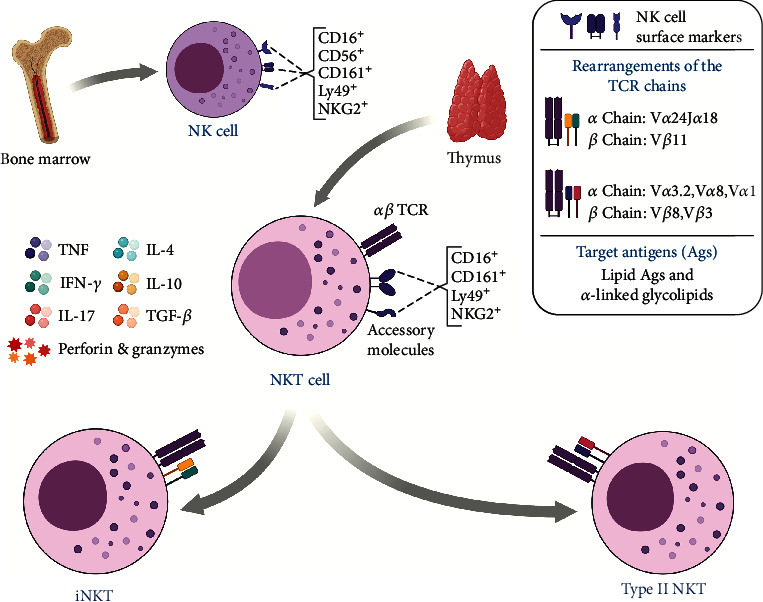
Overview of subpopulations, important receptors, and cytokines produced by NKT cells. Unlike conventional NK cells that mature in the bone marrow, NKT cells develop in the thymus and acquire an invariant or variant TCR, through which their subgroups are stratified. Recent studies show that these lymphocytes participate in the antitumor immune response by recognizing CD1d+ tumors and secreting Th1, Th2, and Th17 profile cytokines.

In regards to their antitumor activity, NKT cells can act directly through cell-cell interaction, through the Fas receptor and its ligand (FasL) that trigger the activation of caspase enzymes and cause apoptosis of the target cells, in addition to the interaction of other receptors such as NKG2D, TRAIL, natural cytotoxicity receptors (NCRs), and their ligands (Figure 2) [111, 113, 122]. There is also the substantial release of perforins and granzymes A and B, in addition to the production of Granulysin that act in a similar way to perforins, forming pores in the plasma membrane and altering their permeability, which results in cell lysis [123, 124].

NKT cells also can act through indirect mechanisms by releasing a range of mediators, especially Th1 or Th2 profile cytokines, which can vary depending on the NKT subtype, a fact that will be discussed later [125]. The release of the aforementioned mediators can results in the immunoregulation of other cells of the immune system, for example, activating or inducing the maturation of DCs through interaction with CD1d or CD40/CD40L and IFN, respectively. After activation of DCs, they will be regulated positively, expressing costimulatory molecules such as CD86 and CD80, in addition to release cytokines such as IL-12, a pleiotropic cytokine that plays an essential role in Th1-type immune response against cancer. iNKT cells also activate CD8 cytotoxic T cells through the IFN, as well as conventional NK cells and macrophages that can act against cancer cells [125–128]. In addition, NKT cells can also stimulate B lymphocytes and induce increased secretion of IgG class antibodies, which can result in antibody-dependent cell cytotoxicity (ADCC) in cancer cells that will subsequently undergo cytotoxic cell-mediated cell lysis [111, 129].

Due to their secretory repertoire and the ability to activate and target other cells of the immune system, NKT cells contribute strongly to tumor immunovigilance. It is important to highlight that one of the main forms of recognition of cancer cells by NKT cells occurs through the interaction of the TCR with the antigen presented by the CD1d molecule, expressed by B cells, macrophages, DCs, and several types of cancer cells, in solid and hematological neoplasms [116, 117]. These cells have been shown to contribute to tumor surveillance and suppression, controlling the initial stage of the neoplastic process [130–133].

iNKT cells, also known as natural killer type I cells, have characteristics that differ from other subsets, such as reactivity to ∝-galactosylceramide (*α*-GalCer) and the more pronounced expression of the Th1 response profile [134]. Another characteristic of iNKT cells is related to its invariant TCR, represented in humans by a TCR-*α* chain (V*α*24J*α*18) and a TCR-*β* chain (V*β*11). iNKT cells recognize lipid antigens and glycolipids such as *α*-GalCer analogs, phospholipids, diacylglycerols, gangliosides (GD3), and glycosphingolipids [107, 112].

Studies show that iNKT cells are strongly reactive to *α*-GalCer, a synthetic glycolipid derived from the marine sponge *Agelas mauritianus*, which is capable of inducing an important immunomodulatory effect in iNKT cells and which stimulates the antitumor response mediated by cytotoxic cells (NK cells and TCD8^+^ lymphocytes) that are in a state of exhaustion or anergy resulting from the TME. Stimulation mediated by iNKT cells has been shown to promote the invigoration of cytotoxic cells and reverse the dysfunction presented by these cells [107, 135].

The *α*-GalCer antigen presented through the CD1d molecule is recognized by the TCR expressed in iNKT cells and induces the production of cytokines, such as IL-2, IL-12, and IL-21, which act on conventional NK cells and exhausted or anergic CD8 cytotoxic T cells, thus, reversing the dysfunction and the hyporesponsive character [125, 136]. In addition, *α*-GalCer-stimulated iNKT cells have been shown to incite the activation of APCs via CD40-CD40L signaling and also induce the production of IL-12 [125, 126, 129, 137].

A study by Fais et al., in patients with acute leukemia, evaluated the expression of the CD1d molecule in LCs, as well as the activity of iNKT cells in their recognition. Where, through incubating LCs with 100 ng/ml of *α*-GalCer for 4 hours, it was observed that the CD1d^+^ LCs associated with the *α*-GalCer antigen underwent apoptosis induced by iNKT cells, in addition to stimulating the production of cytokines INF-*γ*, TNF-*α*, and IL-5 [138]. Similar results were observed by Bojarska-Junak et al. in a study with BP samples from patients with chronic leukemia, in which iNKT cells stimulated *in vitro* for 24 hours with 100 ng/ml of *α*-GalCer showed greater intracellular expression of the INF-*γ* and IL-4 cytokines when compared with healthy volunteers. The study also pointed out that, among the two cytokines analyzed, the expression of IL-4 was greater than that of INF-*γ*, indicating that the iNKT cell may acquire a polarization for the Th2 response profile [139].

Another study showed that the frequency of iNKT cells was lower in patients with chronic leukemia compared to the healthy control group and that therapy for 14 days with IFN-*γ* and 1 *μ*M IM (imatinib mesylate) in combination with 100 ng/ml of *α*-GalCer in these patients resulted in a significant increase in iNKT cells [140]. Similarly, Weinkove et al. demonstrated that LCs purified and stimulated in vitro with 200 ng/ml of *α*-GalCer for 5 days incited the proliferation of autologous and allogeneic iNKT cells, but not in a significant amount it was also demonstrated that there was a stimulation of IFN-*γ* production by iNKT cells in leukemic patients. However, it was observed that the prolonged culture of these cells resulted in the polarization of iNKT cells to a Th2 profile and resulted in high levels of cytokines associated with tumor tolerance [141]. Additional investigations should be carried out to identify how stimulation with *α*-GalCer can provide better results, associating the increased frequency of iNKT cells with polarization for a response profile against LCs.

Finally, in a longitudinal analysis carried out over 18 months with 22 patients that had been diagnosed with leukemia or myelodysplasia and who underwent HLA haploidentical stem cell transplantation for iNKT cell reconstitution, the existence of a correlation between the frequency of iNKT cells and disease remission was reported. The observed data showed that 14 patients whose iNKT cells were completely reconstituted showed remission, and 8 patients whose iNKT cells were not reconstituted showed recurrence, thus, associating the frequency of these cells with a better prognosis [142, 143]. Furthermore, the analysis of the frequency of iNKT cells in the circulation and bone marrow compartment of newly diagnosed patients with acute leukemia demonstrated that a low frequency of these cells is associated with a worse prognosis [144].

Unlike iNKT cells, NKT II cells have an *αβ* TCR variant and form diversified rearrangements. They are not reactive to *α*-GalCer [112, 125, 145]; however, they are restricted to CD1d [146] and recognize antigens such as *β*-glucosylceramide (*β*-GlcCer) and sulfatide, the latter being found in the plasma membrane of myelin, in the central nervous system, in the liver, in the pancreas, and in the kidneys [147–149]. Another characteristic of the NKT II cell is related to its response profile, which is more polarized for the Th2 profile [134, 150].

In contrast to studies on iNKT cells, the number of studies on the role of NKT II cells in leukemia is limited. However, some studies report that NKT II can act in a negative way by contributing to the suppression of surveillance and antitumor activity and, in some situations, contributing to neoplastic progression [111, 112, 151]. Such immunosuppression is mediated through the secretion of IL-13, which activates myeloid-derived suppressor cells (MDSCs), and these, consequently, suppress the activity of tumor-infiltrating cytotoxic cells [150]. It is also believed that this negative regulation of NKT II in the tumor occurs due to a cross-regulation between iNKT and NKT II, where the effect of Th2 cytokines produced by NKT II cells overlap the effect by the Th1 cytokines produced by iNKT, resulting in an immunosuppressed microenvironment. In addition, it has been observed that tumors grow faster when the frequency of NKT II cells is higher than that of iNKT cells, although this suppression mechanism still needs further investigation [111, 134].

It is important to note that, in addition to NKT cells, there are other populations of nonconventional T cells restricted to molecules of the CD1 family, more specifically group 1, composed of CD1a, CD1b, and CD1c, which have lipid antigens or glycolipids, of microbial origin or from the organism itself [152]. Similar to NKT cells, these CD1-restricted cell populations are considered an attractive target for studies in the field of cancer immunotherapy, especially in the context of hematological neoplasms, due to the diversified distribution of CD1 isotypes, where they have been shown to be expressed (CD1a, CD1b, and CD1c) in 75% of acute leukemia blasts [153].

### 2.3. Mucosal-Associated Invariant T (MAIT) Cells

MAIT cells correspond to a population of innate-like T cells and are characterized by the expression of a restricted TCR-*α* with a unique gene rearrangement pattern, namely, TRAV1-2-TRAJ33 / 12/20 (V*α*7.2-J*α*33 / 12/20 in humans), which pair with a limited repertoire of the TCR-*β* chain, predominantly from the TRBV6 and TRBV20 gene families [154–156], and form a semi-invariant TCR restricted to nonpeptide antigens presented by MHC-related protein 1 (MR1). MR1 is a monomorphic molecule that is highly conserved throughout the evolution of mammals [157, 158] and is capable of presenting metabolites derived from vitamin B2 that is synthesized by a variety of microorganisms for MAIT cells [159, 160].

Like most T cells, MAIT cells develop in the thymus [161] and are positively selected by the cortical thymocytes CD4^+^ CD8^+^ MR1^+^ [161, 162]. After the selection process, they undergo extrathymic maturation and integrate with different tissues [163]. In humans, MAIT cells represent up to 10% of peripheral blood T cells and are found in abundance in mucous tissues, mesenteric lymph nodes, and liver, where they can represent up to 45% of all T cells [164, 165].

**Figure 3:**
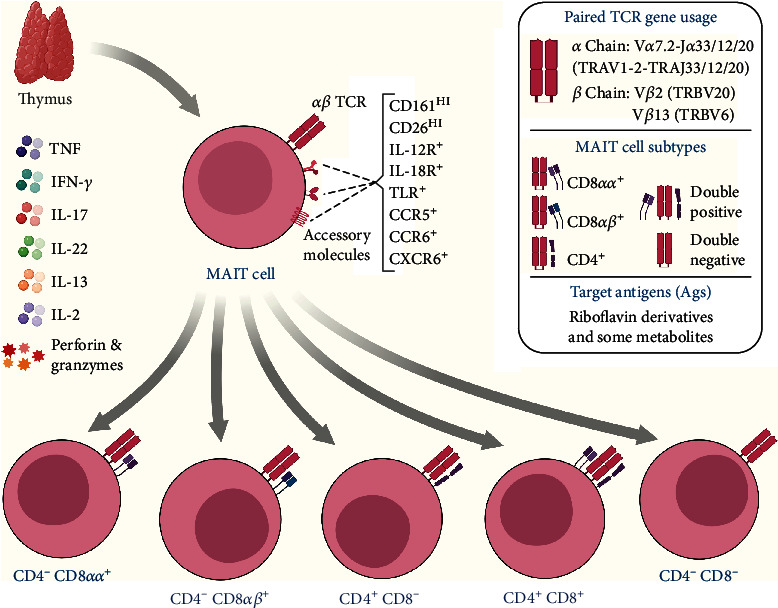
Overview of subpopulations, important receptors, and cytokines produced by MAIT cells. MAIT cells develop in the thymus, where they acquire a semi-invariant TCR, restricted to MR1. In humans, they can be categorized into five subsets based on the expression of the CD4 and CD8 coreceptors, with CD4^−^ CD8*αα*^+^ or CD4^−^ CD8*αβ*^+^ being the most abundant, collectively corresponding to approximately 80% of MAIT cells. They produce a repertoire of Th1 and Th17 cytokines (IFN-*γ*, TNF, IL-2, IL-17A, and IL-22), in addition to perforins and granzymes B, and express various cytokine, chemokine, and homing molecules.

Human MAIT cells can be immunophenotyped as CD3^+^ V*α*7.2^+^ CD161^HI^ [166] and can be categorized based on the expression of CD4 and CD8 coreceptors in five subsets: CD4^+^ CD8^−^, CD4^+^ CD8^+^, CD4^−^ CD8^−^, CD4^−^ CD8*αα*^+^ e CD4^−^ CD8*αβ*^+^ (Figure 3); the last two (CD4^−^ CD8*αα*^+^ and CD4^−^ CD8*αβ*^+^) being the most abundant and collectively correspond to approximately 80% of MAIT cells [164]. Of note, the development of MR1 tetramers loaded with 5-OP-RU [5-(2-oxopropylideneamino)-6-d-ribitylaminouracil] marked a breakthrough in MAIT cell research and have allowed for the reliable identification of distinct phenotypical and functional MAIT cell subsets [167].

In addition to the surface molecules mentioned above, MAIT cells also express CD25, CD26, CD44, and CD69, as well as IL-7R, IL-12R, IL-15R, and IL-18R cytokine receptors and PLZF, T transcription factors T-bet and ROR*γ*t, providing high plasticity and the ability to secrete mediators of Th1 profile and/or Th17 profile [168]. It is important to note that most MAIT cells have a memory-like phenotype effector [169] and can be quickly activated by mechanisms that do not depend on TCR stimulation [170]. This is due to the high expression of various cytokine and chemokine receptors, a property they share with other innate-like T cells (*γδ* and iNKT cells) [171].

After activation, these cells are able to release substantial quantities of perforin and granzymes, in addition to producing the proinflammatory cytokines TNF, IFN*γ*, and IL-17A, as well as CCL3 and CCL4, which are chemokines that are crucial for immune responses to infection and inflammation [172, 173]. The release of these mediators results in the destruction of infected cells and the activation of other immune cells, such as DCs, which consequently leads to the mobilization of conventional T cells and reflects in a cascade of immunological events [174, 175].

Due to some characteristics of MAIT cells, such as their high frequency in humans and their ability to rapidly secrete a repertoire of mediators that induces activation and regulation of other cells of the immune system, in addition to the ability to recognize antigens restricted to MR1, research on MAIT cells, as well as other populations of T cells restricted to MR1, has aroused great interest in the field of oncology. Currently, the functional role triggered by these innate-like T cells in cancer is unclear and has been the subject of several studies.

Investigations performed on patients with mucosal neoplasms have reported a reduction in the frequency of circulating MAIT cells and show a significant accumulation of these cells in the tumor tissue [176–178]. However, it is important to note that tumor-infiltrating MAIT cells were less able to produce IFN-*γ* in response to factors secreted by the TME [178]. In addition, a recent *in vivo* study by Yan et al. showed that MAIT cells exhibited a tumor-promoting function and promoted cancer metastasis through the suppression of cytotoxic cells (which was partly IL-17 dependent) after interaction with MR1 molecules expressed on cancer cells [179].

In contrast, in the study by Won et al., tumor-infiltrating MAIT cells were not compromised in the production of cytokines IFN-y, TNF-*α*, and IL-17. In addition, in the *in vitro* assay, it was observed that MAIT cells, isolated from PB from healthy individuals, not only had lymphokine-activated killer activity but also exhibited direct cytotoxicity in K562 cell lines through the degranulation of granzyme B and perforin [177].

To date, the involvement of MAIT cells in leukemia remains largely unexplored, and, in the context of other hematological neoplasms, the functional role of these cells is also limited. An *in vitro* study by Gherardin et al. demonstrated that multiple myeloma (MM) cell lines express the MR1 protein and are capable of presenting vitamin B metabolites to MAIT cells isolated from healthy donors, which, in response, induce the lysis of these myeloma cells with efficiency and kinetics similar to NK cells [164].

Another study observed the functional capacity of these lymphocytes by analyzing the cell phenotype in samples of PB and BM [180]. Favreau et al. reported that patients recently diagnosed with MM had a significant decrease in the frequency of MAIT cells when compared to healthy controls. They also highlighted a reduction in the MAIT CD8^+^ and double-negative (CD8^−^ CD4^−^) phenotype, in addition to the functional impairment of the Th1 response profile, with fewer IFN-*γ* and TNF*α* producing MAIT cells [180]. Favreau et al. also demonstrated that circulating MAIT cells exhibited high levels of PD-1 and that their respective *in vitro* blockade resulted in the restoration of MAIT cell function and activation [180]. As a whole, these results highlight the important antitumor activity of MAIT cells and identify them as a potential immunotherapeutic target in MM.

Likewise, MR1 also represents an attractive target in immunotherapy against cancer, due to characteristics such as its monomorphic nature and functional expression found in several types of cancer cells [181]. In this regard, a recent study by Crowther et al. demonstrated that a human T cell clone potentially recognizes a specific cancer or associated metabolite, restricted to MR1, and mediates the lysis of different types of cancer cells, including LCs lineages, as such, it mediated *in vivo* leukemia regression and conferred longer survival in mice [182]. Moreover, other atypical MR1-restricted T cells, which respond to autoantigens, have been described [181, 183, 184]. This new group of T cells has been named MR1T and has been shown to recognize and eliminate a wide range of cancer cells that express MR1 [181]. However, more research is needed to elucidate the precise nature and function of these cells, as well as their activity in the context of TME.

## 3. Concluding Remarks and Perspectives

Unconventional T cells can promote tumor rejection and offer advantages that indicate them as being potential targets for T-cell-based immunotherapy. Although effective, it is important to note that unconventional T cells are not exempt from the influence of checkpoint receptors, since these cells positively regulate the inhibitory PD1 receptor on their cell surface after activation [185–187]. However, checkpoint blockade therapy using drugs based on anti-PD1 and anti-CTLA-4 is proving to be a powerful approach for preventing effector cells from entering into a state of anergy caused by cancer cells, thereby, providing a persistent immune response [188].

In the context of hematologic neoplasms, among the promising alternatives to conventional chemotherapy are the upcoming immunotherapies, in particular, the transfer of chimeric antigen receptor (CAR) T cells [189]. These autologous T cells, which are designed to express a CAR receptor against the CD19 antigen, are at the forefront of contemporary oncohematological therapies and lead to high rates of remission in B cell malignancies [190, 191]. It is important to highlight the obstacles, such as the complex and expensive individualized manufacturing process and the loss or modulation of the target CD19 antigen, that lead to resistance and relapse after therapy with CAR T cells.

In this scenario, unconventional T cells present themselves as possible solutions to overcome these obstacles, since they have a series of specific biological characteristics that can significantly expand and diversify the repertoire of CAR-based therapies, although they also have their limitations [192]. Few preclinical studies have investigated unconventional T cells as alternative platforms for CAR engineering, and, given this limitation, it is not surprising that there is a very low number of clinical trials that evaluate the viability of CAR *γδ* T therapies (B cell lymphoma, NCT02656147; and acute myeloid leukemia, NCT03885076) and CAR NKT (refractory B cell neoplasm, NCT03774654) in hematologic neoplasms that are in progress.

The fact is that the role of unconventional T cells during the neoplastic process is usually related to better immune surveillance or antitumor response in patients with leukemia, and that, to date, unconventional T cells are still largely underexplored. Factors, such as the absence of barriers related to histocompatibility, since the molecules that present Ag for unconventional T cells are monomorphic; activation by TCR dependent and independent mechanisms; ability to gather quick and powerful responses; in addition to the high frequency in specific tissues in humans, all demonstrate the need for further studies on the immunotherapeutic potential of these cells and, mainly, the translation of these studies into clinical trials.

## Figures and Tables

**Table 1:** The table indicates some functions triggered by unconventional T cells in the immune response against the tumor.

Subsets	Role played in the immune response against cancer cells	Reference
*γδ* T cells	Mediate tumor regression by recognizing MIC-A/MIC-B and ULBPs through TCR and NKG2D	[34, 35, 51, 52, 90]
	Antitumor potential increased by expression of NCRs	[36]
	Positive regulation of pAgs in LCs mediates the immune response through *γδ* TCR	[40, 41, 72]
	BTN3A/BTN2A1 increases the antitumor functions of *γδ* T cells in blood	[72–74]
	Mediate tumor regression by recognizing PVR and nectin-2 through TCR and DNAM-1	[41]
	BTN3A-expressing LCs are recognized and destroyed by *γδ* T cells in blood through TCR	[69]
	Induce the maturation of DCs, which consequently enhance their activity against LCs	[29, 30]
NKT cells	Mediates tumor regression by recognizing CD1d^+^ LCs through TCR	[133]
	Induces direct destruction of tumor cells through granulysin	[124, 125]
	Induces direct tumor lysis through FasL	[111]
	They act in the immunovigilance during the initial phase of the neoplastic process	[131–134]
MAIT cells	Induces direct cytotoxicity mediated by granzyme B and perforin in cancer cells that express MR1	[162, 175]

## Abbreviations

AICD: Activation-induced cell death
ALL: Acute lymphoblastic leukemia
AML: Acute myeloid leukemia
ANX-2: Annexin-2
APCs: Antigen-presenting cells
BM: Bone marrow
BTN: Butyrophilin
BTN2A1: Butyrophilin subfamily 2 member A1
BTN3A: Butyrophilin 3A
CAR: Chimeric antigen receptor
CD: Cluster of differentiation
CDR3: Complementarity-determining region–3
CTLA-4: Cytotoxic T-lymphocyte–associated antigen 4
CLL: Chronic lymphocytic leukemia
CML: Chronic myeloid leukemia
DCs: Dendritic cells
DNAM-1: DNAX accessory molecule-1
Fas: Fas Cell Surface Death Receptor
FasL: Fas ligand
Fc*γ*RIII: Fc-gamma receptor type III
GM-CSF: Granulocyte-macrophage colony-stimulating factor
HIV: Human immunodeficiency virus
HMB-PP: (E)-4-Hydroxy-3-methyl-but-2-enyl pyrophosphate
IFN: Interferon
IL: Interleukin
iNKT: Invariant NKT
IPP: *Isopentenyl pyrophosphate*
LCs: Leukemic cells
MAIT: Mucosal-associated invariant T
MHC: Major histocompatibility complex
MIC-A: MHC class I polypeptide-related sequence A
MIC-B: MHC class I polypeptide-related sequence B
MM: Multiple myeloma
MR1: MHC class I-related protein
NCRs: Natural cytotoxicity receptors
NK: Natural killer
NKG2D: Natural-killer group 2 member D
NKT: Natural killer T
PB: Peripheral blood
PD1: Programmed cell death protein 1
pAgs: Phosphoantigens
PVR: Poliovirus receptor
TCR: T-cell receptor
TGF: Transforming growth factor
TLRs: Toll-like receptors
TME: Tumor microenvironment
TNF: Tumor necrosis factor
TRAIL: TNF-related apoptosis-inducing ligand
ULBP: UL16-binding protein
*α*-GalCer: *α*-Galactosylceramide
*γδ* T: Gamma-delta T.

## Additional Points

Cell-mediated immune response in malignant neoplasms is becoming the focus in immunotherapy against cancer. Unconventional T cells utilize a range of receptors and proinflammatory or regulatory profiles that directly contribute to the immune response in the leukemic microenvironment. The role played by unconventional T cells found in the leukemic medullary microenvironment may be related to the patient's prognosis. Cytokines and chemokines secreted by unconventional T cells include IL-1*β*, IL-4, IL-6, IL-10, IL-13, IL-17, IL-22, IL-23, IFN-*γ*, TNF-*α*, and TGF-*β*.
